# Advancing metagenome-assembled genome-based pathogen identification: unraveling the power of long-read assembly algorithms in Oxford Nanopore sequencing

**DOI:** 10.1128/spectrum.00117-24

**Published:** 2024-04-30

**Authors:** Zhao Chen, Christopher J. Grim, Padmini Ramachandran, Jianghong Meng

**Affiliations:** 1 Joint Institute for Food Safety and Applied Nutrition, Center for Food Safety and Security Systems, University of Maryland, College Park, Maryland, USA; 2 Center for Food Safety and Applied Nutrition, United States Food and Drug Administration, College Park, Maryland, USA; 3 Department of Nutrition and Food Science, University of Maryland, College Park, Maryland, USA; The Pennsylvania State University Food Science, University Park, Pennsylvania, USA

**Keywords:** metagenome assembly, Oxford Nanopore sequencing, long read, nanopore chemistry, sequencing depth, metagenome-assembled genome, pathogen identification

## Abstract

Oxford Nanopore sequencing is one of the high-throughput sequencing technologies that facilitates the reconstruction of metagenome-assembled genomes (MAGs). This study aimed to assess the potential of long-read assembly algorithms in Oxford Nanopore sequencing to enhance the MAG-based identification of bacterial pathogens using both simulated and mock communities. Simulated communities were generated to mimic those on fresh spinach and in surface water. Long reads were produced using R9.4.1+SQK-LSK109 and R10.4 + SQK-LSK112, with 0.5, 1, and 2 million reads. The simulated bacterial communities included multidrug-resistant *Salmonella enterica* serotypes Heidelberg, Montevideo, and Typhimurium in the fresh spinach community individually or in combination, as well as multidrug-resistant *Pseudomonas aeruginosa* in the surface water community. Real data sets of the ZymoBIOMICS HMW DNA Standard were also studied. A bioinformatic pipeline (MAGenie, freely available at https://github.com/jackchen129/MAGenie) that combines metagenome assembly, taxonomic classification, and sequence extraction was developed to reconstruct draft MAGs from metagenome assemblies. Five assemblers were evaluated based on a series of genomic analyses. Overall, Flye outperformed the other assemblers, followed by Shasta, Raven, and Unicycler, while Canu performed least effectively. In some instances, the extracted sequences resulted in draft MAGs and provided the locations and structures of antimicrobial resistance genes and mobile genetic elements. Our study showcases the viability of utilizing the extracted sequences for precise phylogenetic inference, as demonstrated by the consistent alignment of phylogenetic topology between the reference genome and the extracted sequences. R9.4.1+SQK-LSK109 was more effective in most cases than R10.4+SQK-LSK112, and greater sequencing depths generally led to more accurate results.

**IMPORTANCE:**

By examining diverse bacterial communities, particularly those housing multiple *Salmonella enterica* serotypes, this study holds significance in uncovering the potential of long-read assembly algorithms to improve metagenome-assembled genome (MAG)-based pathogen identification through Oxford Nanopore sequencing. Our research demonstrates that long-read assembly stands out as a promising avenue for boosting precision in MAG-based pathogen identification, thus advancing the development of more robust surveillance measures. The findings also support ongoing endeavors to fine-tune a bioinformatic pipeline for accurate pathogen identification within complex metagenomic samples.

## INTRODUCTION

Culture-independent shotgun metagenomic sequencing has brought about a significant transformation in our comprehension of complex microbiomes across various microbiological domains (1). Since the groundbreaking reconstruction of the first near-complete metagenome-assembled genomes (MAGs) in 2004 (2), genome-resolved metagenomics has played a crucial role in advancing food safety and public health efforts. MAGs enable the identification and monitoring of potential pathogenic microorganisms in food and environmental samples. By assembling genomes from metagenomic data, it becomes possible to discern the presence of specific pathogens, aiding in the early detection of potential risks. MAGs offer a higher resolution at the strain level, allowing for the differentiation of closely related bacterial strains (3). Moreover, MAGs facilitate the identification of antimicrobial resistance (AMR) genes (ARGs) within microbial communities associated with food and environment (4). This is essential for monitoring and addressing the emergence of AMR, guiding the development of strategies to mitigate risks associated with antimicrobial-resistant strains.

Traditionally, high-throughput Illumina sequencing has been the primary choice for shotgun metagenomic investigations (5). Although Illumina reads offer high accuracy (~0.1%–0.5% error rate), their limitation lies in their short lengths, typically ranging from 50 to 300 bp (6). During *de novo* assembly, Illumina sequencing often results in fragmented assemblies due to its inability to assemble genomic regions containing repetitive elements longer than library insert sizes and read lengths (7). Even when striving for high-quality MAGs using Illumina short reads, assemblies can remain highly fragmented, potentially missing crucial genetic information (8). This issue becomes particularly pronounced in food and environmental microbiomes containing closely related species or sub-species with lengthy repetitive regions of unknown abundance. Such scenarios can lead to extensive homologies between genomes, hindering the assembly process (9).

Third-generation sequencing has emerged as a solution to tackle the challenges posed by ambiguous repetitive regions and to enhance the continuity of genome assemblies (10). Among these innovations, Oxford Nanopore Technologies, Inc. (ONT) has developed nanopore-based sequencing, which allows for direct, real-time analysis of long DNA fragments and has found wide-ranging applications (11). Despite nanopore sequencing being associated with error-prone reads (with an error rate exceeding 1%), it has demonstrated a remarkable capability to generate highly complete genomes, which has sparked a renaissance in the reconstruction of MAGs from diverse food microbiomes (12–15).

In recent years, the R9 chemistry has been extensively employed for Oxford Nanopore sequencing (16). However, due to the limitation of having only one reader head, the R9 chemistry faces challenges in accurately identifying bases within certain sequences, particularly insertions and deletions (indels) originating from homopolymeric regions with continuous identical bases (17, 18). To address this issue, ONT introduced the R10 chemistry in late 2021, featuring a longer barrel and dual reader heads (19). This upgrade has enhanced the resolution of homopolymeric regions and improved the consensus accuracy of nanopore sequencing data.

**Fig 1:**
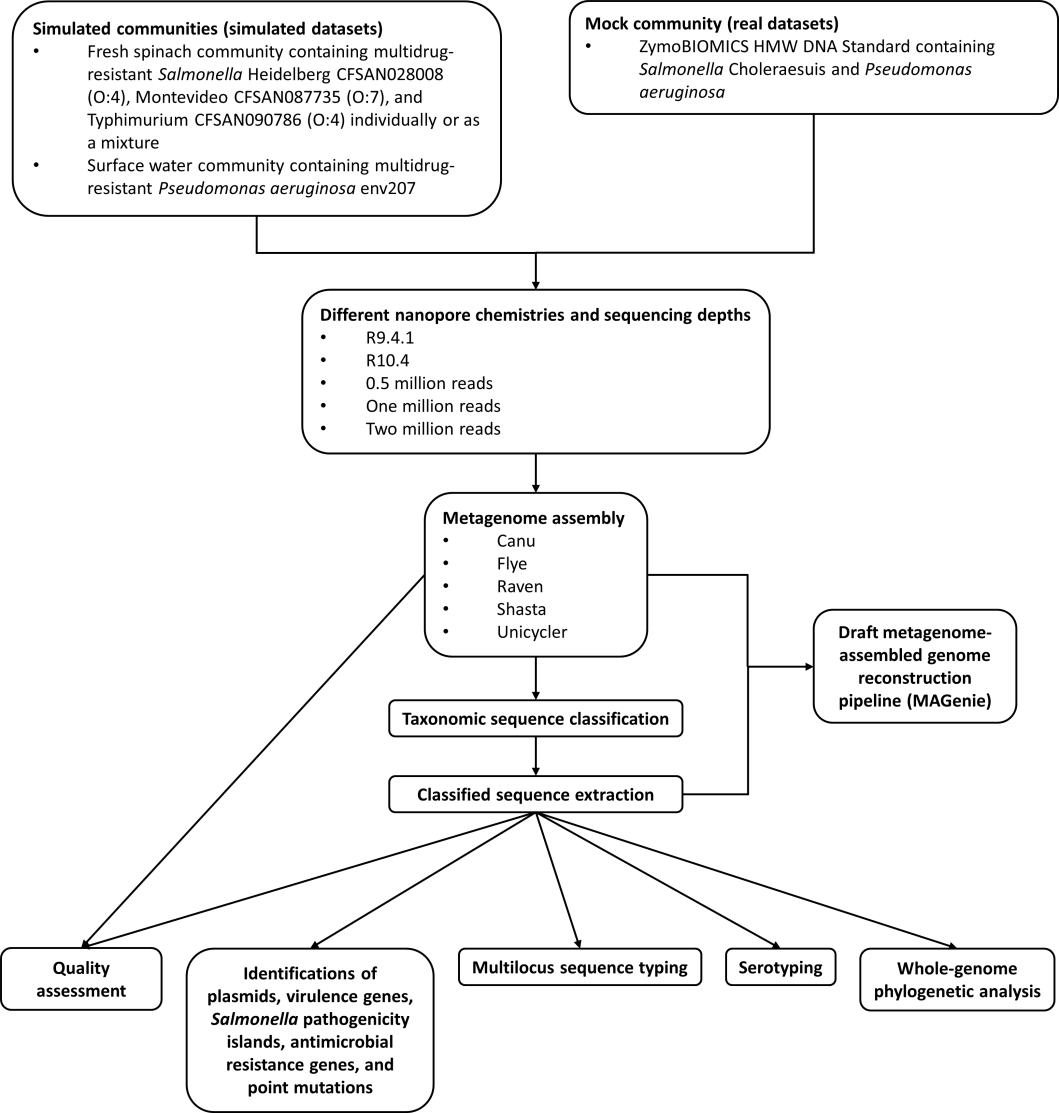
A comprehensive flow chart detailing the step-by-step methodology employed in the study and the bioinformatic pipeline (MAGenie) that combines metagenome assembly, taxonomic classification, and sequence extraction to reconstruct draft metagenome-assembled genomes.

Metagenomic sequencing technologies and associated bioinformatic tools are often evaluated using simulated and mock communities, where the relative abundance of each microorganism is precisely known. However, given the rapid evolution of assembly algorithms designed for Oxford Nanopore long reads, there has been limited investigation into whether the current array of available long-read assemblers can enhance subsequent identification of bacterial pathogens in metagenomic data. In this study, we sought to address this gap by employing both simulated communities (representing bacterial populations found on fresh spinach and in surface water) and real metagenomic data sets (Fig. 1). Our comprehensive assessment focused on determining the efficacy of state-of-the-art long-read assemblers tailored for Oxford Nanopore sequencing within a metagenomic context with the presence of foodborne and waterborne pathogens. A primary emphasis was placed on evaluating whether each assembly process could yield complete and accurate assemblies for downstream genomic analyses. Our study represents the first critical assessment of long-read assemblers for Oxford Nanopore sequencing when applied to complex microbiomes to identify bacterial pathogens. We also developed a bioinformatic pipeline to reconstruct draft MAGs of bacterial pathogens from metagenome assemblies.

## MATERIALS AND METHODS

### Simulated data sets

**Fig 2:**
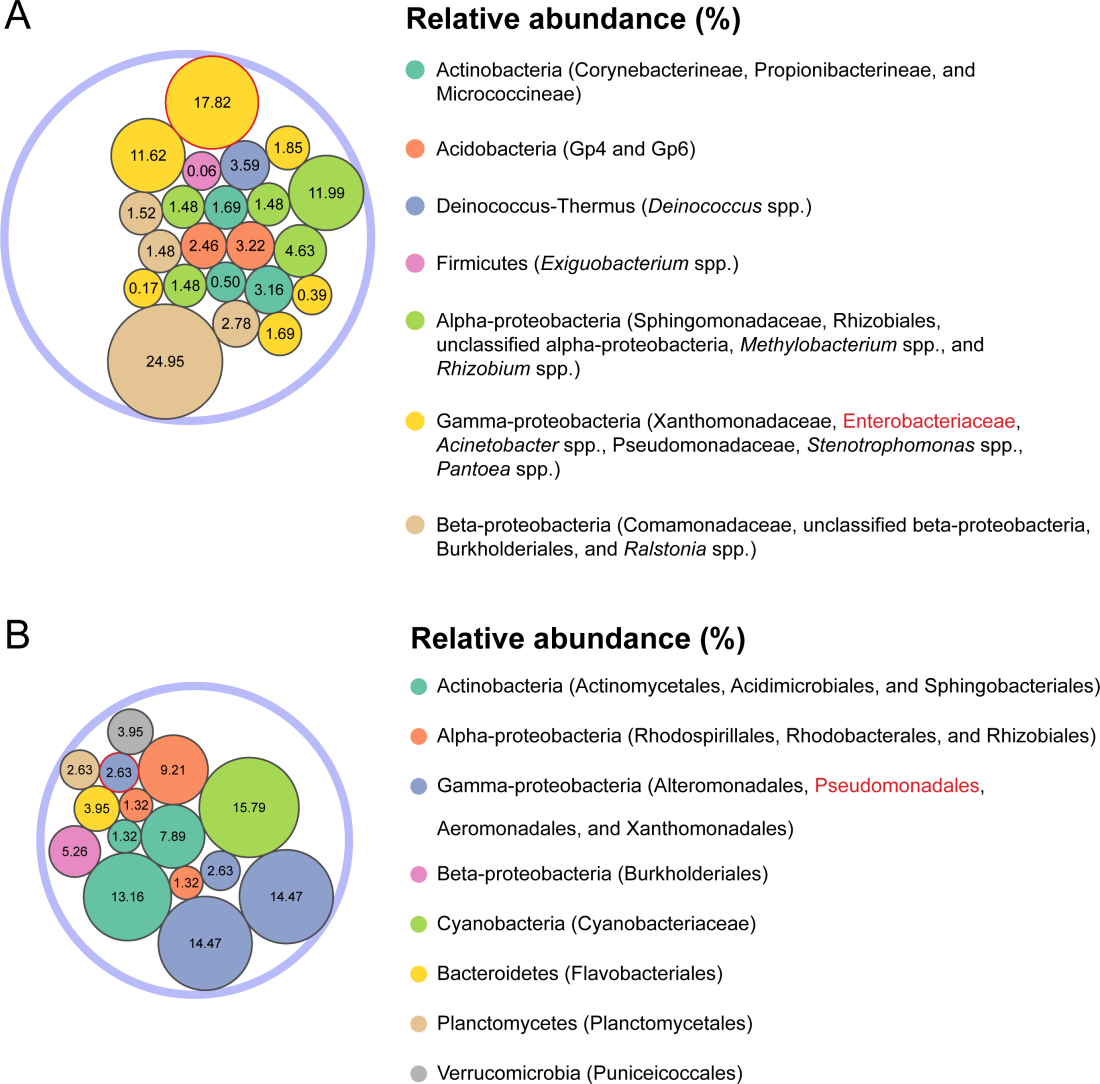
Relative abundance of each family or order in the simulated bacterial communities on fresh spinach (**A**) and in surface water (**B**). Enterobacteriaceae and Pseudomonadales *Salmonella enterica* and *Pseudomonas aeruginosa* represent, respectively, are indicated in circles with red edge. The size of each circle is proportional to the relative abundance of the family or order it represents.

The simulated bacterial community on fresh spinach was designed based on the relative abundances reported by Lopez-Velasco et al. (20). This community encompassed five phyla: Acidobacteria, Actinobacteria, Deinococcus‐thermus, Firmicutes, and Proteobacteria (comprising alpha-proteobacteria, gamma-proteobacteria, and beta-proteobacteria) (Fig. 2A; Table S1). To represent the family of Enterobacteriaceae, multidrug-resistant *Salmonella enterica* serotype Heidelberg CFSAN028008, Montevideo CFSAN087735, or Typhimurium CFSAN090786 was individually included in the fresh spinach community. To investigate the impact of a mixture of three serotypes on metagenomic identification, we combined genomes of *S*. Heidelberg, Montevideo, and Typhimurium, all with equal relative abundance, representing the family of Enterobacteriaceae. Notably, *S*. Heidelberg and Typhimurium were both part of group O:4, while Montevideo belonged to group O:7 (21). In the case of the simulated bacterial community in surface water (urban river water), it was formulated according to Beale et al. (22) to consist of six phyla: Acidobacteria, Actinobacteria, Bacteroidetes, Planctomycetes, Proteobacteria (encompassing alpha-proteobacteria, gamma-proteobacteria, and beta-proteobacteria), and Verrucomicrobia (Fig. 2B; Table S2). To represent the order of Pseudomonadales, multidrug-resistant *Pseudomonas aeruginosa* env207 was included in the surface water community (Table S2). The relative abundance of each family was standardized to ensure that the total relative abundance of the bacterial community equaled 100%, focusing exclusively on classified reads while excluding unclassified and eukaryotic reads. The relative abundance circular packing plot of each simulated bacterial community was generated using “packcircles” 0.3.6 (23) and “ggplot2” 3.4.4 (24) R packages (R 4.3.2).

To assess the impact of different nanopore chemistries on metagenome assembly, we initiated the characterization stage of Nanosim 3.1.0 (25). This stage involved training and building error models based on the real R9.4.1 and R10.4 reads of the ZymoBIOMICS HMW DNA standard (Zymo Research Corporation, Irvine, CA) (17) (Table S3). The reads were basecalled in the super-accurate mode using Guppy 5.0.16. Utilizing minimap2 for read alignment, Nanosim employs a list of reference genomes and a training metagenomic data set to construct an alignment-based error model and generate a collection of read characterization profiles for the subsequent simulation stage. In the simulation stage, we employed NanoSim to generate Oxford Nanopore long reads using the error models derived from the characterization stage. This process utilized Guppy as the basecaller to simulate homopolymers and/or base qualities. Additionally, a minimum homopolymer length of 6 (*k*-mer bias) was applied to replicate homopolymer contraction and expansion events. To investigate the impact of sequencing depth on metagenome assembly, we initially generated 0.5 million reads from each error model to simulate the bacterial communities on fresh spinach and in surface water. Subsequently, we produced 1 and 2 million reads to explore whether higher sequencing depths could enhance downstream genomic analyses.

### Real data sets

We sourced real data sets from the study conducted by Sereika et al. (17), which comprised high-coverage Oxford Nanopore long reads obtained from the ZymoBIOMICS HMW DNA Standard (Table S3). This mock community was composed of a diverse mix of high-molecular-weight genomic DNA from seven bacterial (*S*. Choleraesuis, *P. aeruginosa*, *Escherichia coli*, *Enterococcus faecalis*, *Staphylococcus aureus*, *Listeria monocytogenes*, and *Bacillus subtilis*) and one fungal genomes (*Saccharomyces cerevisiae*). The distribution of these organisms within the community was uniform, with each bacteria representing 14% and *S. cerevisiae* 2% of the total composition. For our research, we focused on studying *S*. Choleraesuis and *P. aeruginosa* from the ZymoBIOMICS HMW DNA Standard. The data sets R9.4.1 and R10.4 were generated utilizing the R9.4.1 flow cell with SQK-LSK109 (R9.4.1+SQK-LSK109) and the R10.4 flow cell with SQK-LSK112 (R10.4 + SQK-LSK112) (ONT, Oxford, UK) on MinION and PromethION (ONT), respectively. SQK-LSK112, an upgraded ligation sequencing kit, was specifically optimized for high sequencing accuracy. It incorporates a new sequencing adapter (Adapter Mix H) that enhances accuracy to over 99%. This upgrade also results in lower flow cell loading requirements and introduces fuel fix technology, allowing for extended experimental runs without the need for fuel addition during the sequencing process. For simplicity, we referred to the sequencing runs performed using R9.4.1+SQK-LSK109 as “R9.4.1.” Similarly, the sequencing runs utilizing R10.4+SQK-LSK112 were denoted as “R10.4.” These sequencing processes were carried out using MinKNOW 21.05.25 (ONT). To explore the impact of sequencing depth on metagenome assembly, we randomly sub-sampled 0.5, 1, and 2 million reads from the real data sets (26). The quality of Oxford Nanopore long reads was assessed and scored using NanoPlot 1.0.0 (Table S3; Fig. S1) (27). Sub-sampled reads and raw reads from the real data sets exhibited statistically similar quality characteristics.

### Data pre-processing

The trimming of adapters at the ends of Oxford Nanopore long reads was performed using Porechop 0.2.4 (https://github.com/rrwick/Porechop). In cases where a read contained an adapter in its middle, the read was identified as chimeric and subsequently split into two separate reads, with the adapter removed. NanoFilt 2.8.0 was used to discard reads with lengths below 1,000 bp (27).

### MAGenie: a bioinformatic pipeline to reconstruct draft MAGs

In this study, we developed a bioinformatic pipeline (MAGenie) (https://github.com/jackchen129/MAGenie) to reconstruct draft MAGs, comprising several sequential steps facilitated by publicly available tools, including metagenome assembly, taxonomic classification, and sequence extraction:

1. Metagenome assembly: We benchmarked five widely used long-read assemblers, which included Canu 2.2 (28), Flye 2.9.2 (29), Raven 1.8.1 (30), Shasta 0.10.0 (31), and Unicycler 0.5.0 (32). Canu was run with the average genome size of the reference genomes in each community as the estimated genome size, 1,000 bp as the minimum read length, 500 bp as the minimum overlap, and 40 bp as the target coverage for corrected reads. To assemble with Flye, *k*-mer sizes were automatically computed, with the metagenome assembly mode (metaFlye) and one polishing iteration. Raven was subjected to a *k*-mer size of 15 bp, sliding window length of 5 bp, threshold for ignoring most frequent minimizers of 0.001, maximum number of overlaps of 32, two polishing rounds, score for matching bases of 3, score for mismatching bases of −5, gap penalty of −4, and minimal unitig size of 9,999. Shasta was run with built-in assembly configurations of Nanopore-Phased-May2022 and Nanopore-Phased-R10-Fast-Nov2022 for R9.4.1 and R10.4 chemistries, respectively. The long-read assembly process of Unicycler was performed with normal bridging mode, 3 as the alignment scores of match, −6 as the alignment scores of mismatch, −5 as the alignment scores of gap open, and −2 as the alignment scores of gap extend. The assembly processes of Raven and Shasta were executed on a Linux operating system with Ubuntu 23.04 LTS, utilizing 28 threads of central processing unit and 64 GB of random-access memory. Canu, Flye, and Unicycler were run on GalaxyTrakr, a specialized platform developed by the U.S. Food and Drug Administration (FDA) for use by GenomeTrakr laboratories (33).
2. Taxonomic classification: We employed Kraken 2 2.1.3 (34) for the taxonomic classification of each sequence in our metagenome assemblies. This classification was performed against the standard database (database created: 09/2019), utilizing the following parameters: *k*-mer size, 35 bp; minimizer length, 35 bp; minimizer space, 6 bp; and two minimum hit groups.
3. Sequence extraction: We specifically focused on sequences classified under the following taxonomies: *Salmonella* (taxonomy ID: 590), *S*. Heidelberg (taxonomy ID: 454169), *S*. Montevideo (taxonomy ID: 115981), *S*. Typhimurium (taxonomy ID: 90371), and *Pseudomonas* (taxonomy ID: 286). To extract and compile these sequences from each assembly, we utilized the sequence extraction module of KrakenTools (35). This extraction encompassed reads classified at both parent and child taxonomic levels.

### Quality assessment

We conducted a comprehensive evaluation of the quality of each metagenome assembly using QUAST 5.2.0 (36), augmented with the MetaQUAST extension (37). This evaluation generated various relevant quality metrics, including the number of contigs, total length (bp), the length of the largest contig (bp), N50, and L50. The performance of each assembler was assessed by aligning the extracted sequences, classified as either *Salmonella* or *Pseudomonas*, to their respective reference genomes. Additionally, we computed various quality metrics, including GC content (%), genome fraction (%), and numbers of misassemblies, mismatches, indels, and single-nucleotide polymorphisms (SNPs). To further evaluate the nucleotide-level genomic similarity between the extracted sequences and the reference genomes, the average nucleotide identity (ANI) (%) was calculated using FastANI 1.33 (38). For a quantitative assessment of metagenome and genome completeness, a Benchmarking Universal Single-Copy Ortholog (BUSCO) analysis was performed using BUSCO 5.4.7 (39). This analysis evaluated the expected gene content of each assembly and considered length alignments to the BUSCO profiles. We used a BLAST search with a cutoff *E*-value of 0.001 and considered three candidate regions. The scale of genome completeness was expressed as complete BUSCO (%) representing the fractions of high-identity, full-length genes. For the extracted sequences derived from simulated communities, we employed RefSeq Masher 0.1.2 (40) to screen for potential contamination by other microorganisms in the communities.

### Identifications of plasmids, virulence genes, *Salmonella* pathogenicity islands, ARGs, and point mutations

Plasmids were identified with Staramr 0.9.1 (41) by comparing with known plasmid sequences incorporated with the PlasmidFinder database (42), with 98% minimum identity and 60% minimum coverage. Virulence genes were detected with ABRicate 1.0.0 against known gene sequences (https://github.com/tseemann/abricate) in the Virulence Factors Database (43), with 80% minimum identity and 60% minimum coverage. SPIFinder 2.0 (44) was employed to identify *Salmonella* pathogenicity islands (SPIs), with 95% minimum identity and 60% minimum coverage. ARGs and point mutations were detected using AMRFinderPlus 3.11.14 (45), with −1 minimum identity and 50% minimum coverage. Afterward, to identify if the ARGs originated from the pathogens or other microorganisms in the community, contigs containing ARGs were searched for highly similar sequences in the reference genomes using NCBI BLAST+ 2.14.0 blastn with the MegaBLAST algorithm (46), with 0.001 as the value cutoff. Heatmaps were generated with the “pheatmap” 1.0.12 (47) and “ggplot2” R packages.

### Serotyping

*S. enterica* serotype was predicted from the extracted sequences using SISTR 1.1.2 (48), which relies on Mash MinHash for determining antigen gene and core-genome multilocus sequence typing (MLST) gene alleles (39). Additionally, for the serotyping of *Pseudomonas aeruginosa*, we utilized PAst 1.0 based on Thrane et al. (49), which examines the O-specific antigen for serotyping.

### MLST

MLST was performed using mlst 2.23.0 (50; https://github.com/tseemann/mlst) with integrated components of the PubMLST database. The analysis involved scanning the extracted sequences against traditional PubMLST typing schemes, specifically those based on seven housekeeping genes. We used criteria that included a minimum identity of the full allele at 95%, a minimum coverage of the partial allele at 10%, and a minimum score of 50 to match a scheme. The analysis also provided the assignment of a sequence type (ST) as reported by mlst.

### Whole-genome phylogenetic analysis

To examine how the extracted sequences classified as *Salmonella* performed in the whole-genome phylogenetic inference, they were compared with corresponding reference genomes, 30 *Salmonella* genomes of the same serotype, and 10 *Salmonella* genomes of different serotypes (Tables S4 to S7). For the fresh spinach community containing three serotypes, the extracted sequences for each serotype were also compared with 10 *Salmonella* genomes of two other serotypes (Tables S4 to S6). In comparison, the extracted sequences classified as *Pseudomonas* were compared with corresponding reference genomes, 30 *P*. *aeruginosa* genomes, and 10 *Pseudomonas* genomes of different species (Table S8). Whole-genome SNPs were called using CSI Phylogeny 1.4 based on the concatenated alignment of the high-quality SNP (51), with 10 bp as the minimum distance between SNPs, 30 as the minimum SNP quality, 10× as the minimum depth at SNP positions, 10% as the minimum relative depth at SNP positions, 25 as the minimum read mapping quality, and 1.96 as the minimum *Z*-score. *S*. Heidelberg CVM 35161 (RefSeq assembly accession: GCF_016452005.1), *S*. Montevideo R17.4849 (RefSeq assembly accession: GCF_024266285.1), *S*. Typhimurium LT2 (RefSeq assembly accession: GCF_000006945.2), *S*. Choleraesuis 1633 (RefSeq assembly accession: GCF_018359685.1), and *P. aeruginosa* DSM 50071 (RefSeq assembly accession: GCF_001045685.1) served as the reference genomes to construct the whole-genome phylogenetic trees for *S*. Heidelberg, Montevideo, Typhimurium, Choleraesuis, and *P. aeruginosa*, respectively. Each inferred whole-genome phylogeny was visualized as a rooted rectangular phylogram using iTOL 6.7.4 (52).

### Statistical analyses

To assess if significant differences (*P* < 0.05) existed among different assemblers, nanopore chemistries, and sequencing depths, we conducted pairwise comparisons using *t*-test in R. To account for multiple comparisons, we also employed the Bonferroni correction, adjusting the significance threshold to α/*n*, where α is the initial significance level (0.05), and *n* is the total number of independent comparisons.

## RESULTS

### Quality of metagenome assemblies

Flye, Raven, and Shasta consistently completed the assembly process, while Canu and Unicycler encountered challenges in some cases due to runtime or memory requirements.

#### Simulated fresh spinach community

Flye, Raven, and Unicycler produced assemblies with notably higher total lengths, lengths of the largest contig, and N50 values (Tables S9 to S12). They also typically yielded fewer contigs and lower L50 values compared to Canu and Shasta. All assemblies achieved 100% complete BUSCOs, with only two exceptions found in the Shasta assemblies of R10.4 reads from the community containing *S*. Heidelberg or Typhimurium, where complete BUSCO fell below 100%. R9.4.1 and R10.4 reads tended to result in assemblies with similar quality. Higher sequencing depths led to assemblies with increased total lengths, lengths of the largest contig, N50 values, and complete BUSCOs.

#### Simulated surface water community

The Flye assemblies exhibited superior characteristics, including greater total lengths and N50 values (Table S13). However, they also resulted in fewer contigs and lower L50 values compared to assemblies produced by other assemblers. Following Flye, Raven and Unicycler performed reasonably well, while Shasta proved less effective in generating high-quality assemblies. A noteworthy pattern emerged when comparing assemblies of R10.4 reads to those from R9.4.1 reads, with R10.4 reads leading to assemblies with higher numbers of contigs and L50 values but reduced total lengths, lengths of the largest contig, and N50 values in most cases. Higher sequencing depths were associated with assemblies featuring greater total lengths, lengths of the largest contig, and N50 values. Importantly, all assemblies achieved 100% complete BUSCOs.

#### ZymoBIOMICS HMW DNA standard

The Flye assemblies consistently outperformed other assemblers, yielding assemblies with greater total lengths, lengths of the largest contig, and N50 values (Table S14). Notably, they produced fewer contigs compared to the assemblies generated by other assemblers. Following Flye, in terms of assembly quality, Raven, Unicycler, and Shasta ranked in descending order, while Canu produced assemblies of lower quality. All assemblies, however, exhibited similar L50 values and achieved 100% complete BUSCOs. The assemblies of R9.4.1 reads performed similarly to those of R10.4 reads. In general, different sequencing depths resulted in similar performance.

### Quality of the extracted sequences

#### Simulated fresh spinach community

In the case of the community containing only one serotype, draft MAGs were successfully obtained in some instances (Tables S15 to S17). Notably, the Flye assemblies of 0.5 and 1 million R9.4.1 reads, as well as 1 and 2 million R10.4 reads in the community containing *S*. Montevideo, and 0.5 and 1 million R9.4.1 reads, along with 0.5 million R10.4 reads in the community containing *S*. Typhimurium, produced particularly promising results. The Flye assemblies consistently demonstrated higher assembly quality compared to other assemblers, with greater total lengths, lengths of the largest contig, N50 values, genome fractions, and ANIs. However, they exhibited lower L50 values and fewer misassemblies, mismatches, indels, and SNPs. In terms of assembly quality rankings, Raven, Shasta, and Unicycler performed moderately, while Canu showed limitations in generating high-quality assemblies. Furthermore, sequences extracted from the assemblies of R9.4.1 reads had greater lengths of the largest contig but fewer misassemblies compared to R10.4 reads. Higher sequencing depths appeared to show greater total lengths, lengths of the largest contig, N50, and genome fractions but lower L50 values and fewer misassemblies compared to R10.4 reads.

When we introduced three serotypes into the community, we were unable to acquire draft MAGs using the extracted sequences from the assemblies, regardless of assembler, nanopore chemistry, or sequencing depth (Tables S18 to S20). The Raven assemblies exhibited longer total lengths and larger contig lengths but fewer contigs, along with fewer mismatches, indels, and SNPs. Notably, sequences extracted from the assemblies of R9.4.1 and R10.4 reads displayed similar quality in most cases, but the extracted sequences classified as *S*. Heidelberg from the assemblies of R9.4.1 reads showed higher genome fractions than those of R10.4 reads. In general, varying sequencing depths yielded assemblies of comparable quality. However, increased sequencing depths correlated with higher L50 values, specifically for the extracted sequences that were classified as *S*. Montevideo.

#### Simulated surface water community

We achieved a draft MAG using the Flye assembly of 2 million R10.4 reads (Table S21). Flye outperformed other assemblers, displaying superior assembly quality characterized by greater total lengths, lengths of the largest contig, N50 values, and genome fractions but fewer SNPs. Raven, Shasta, and Unicycler performed moderately. No significant differences were observed between sequences extracted from the assemblies of R9.4.1 and R10.4 reads. Higher sequencing depths resulted in assemblies of higher quality, characterized by increased number of contigs, total lengths, lengths of the largest contig, N50 values, L50 values, genome fractions, and ANIs, along with decreased mismatches and indels.

For the extracted sequences derived from both simulated communities, the results from RefSeq Masher unequivocally confirmed the absence of contamination by other microorganisms in the communities.

#### ZymoBIOMICS HMW DNA standard

When analyzing the extracted sequences classified as *Salmonella*, we achieved draft MAGs with the Flye assemblies of 0.5 and 2 million R9.4.1 reads, the Shasta assemblies of 0.5 and 1 million R9.4.1 and R10.4 reads, and the Unicycler assembly of 1 million R9.4.1 reads (Table S22). As an example, sequences extracted from the Shasta assembly of 1 million R10.4 reads yielded a draft MAG with a size of 4,778,133 bp, comprising only three contigs. The length of the largest contig was 4,751,113 bp, closely matching the genome size of the reference genome (4,759,746 bp). In terms of overall performance, Flye and Unicycler showed similar effectiveness, followed by Raven and Shasta, while Canu demonstrated the least favorable outcomes. Sequences extracted from the assemblies of R9.4.1 and R10.4 reads exhibited similar quality. Increasing sequencing depths led to an increase in complete BUSCOs.

When examining the extracted sequences classified as *P. aeruginosa*, we successfully obtained draft MAGs through various assemblies (Table S23). Flye consistently produced draft MAGs, as did the Raven assemblies of 0.5 and 1 million R9.4.1 reads, and 1 and 2 million R10.4 reads, the Shasta assemblies of 0.5, 1, and 2 million R9.4.1 reads, and 2 million R10.4 reads, along with the Unicycler assemblies of 0.5 and 1 million R9.4.1 reads and 1 million R10.4 reads. Remarkably, sequences extracted from the Raven assemblies of 0.5 million R9.4.1 and R10.4 reads, the Shasta assembly of 2 million R10.4 reads, and the Unicycler assemblies of 0.5 and 1 million R9.4.1 reads yielded the most contiguous draft MAGs, which comprised only one contig. Overall, Flye, Raven, and Unicycler demonstrated superior performance among the assemblers, closely trailed by Shasta, while Canu displayed less favorable outcomes. Sequences extracted from the assemblies of R9.4.1 reads exhibited higher genome fractions compared to sequences extracted from the assemblies of R10.4 reads. Higher sequencing depths led to a reduction in mismatches.

### Plasmids

#### Simulated fresh spinach community

**Fig 3:**
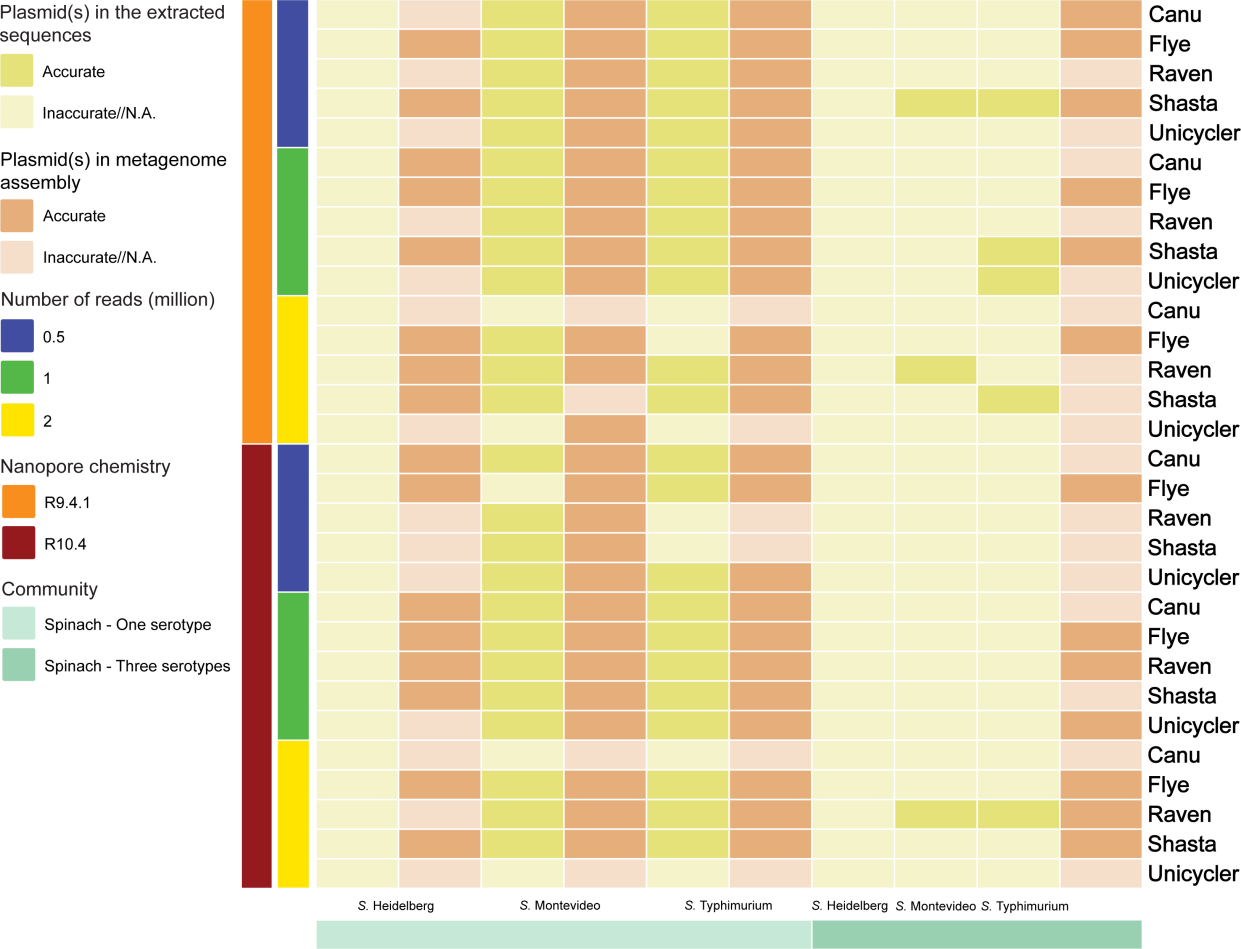
Plasmids in the extracted sequences from metagenome assemblies.

In the community containing *S*. Heidelberg, Flye demonstrated superior performance over other assemblers in terms of plasmid identification, achieving accurate results in all cases (Fig. 3; Table S24). Shasta closely followed Flye in plasmid identification. Canu and Raven displayed similar performance, while Unicycler assemblies did not yield any accurate results in this scenario. However, when dealing with the community containing *S*. Montevideo or Typhimurium, all assemblers performed similarly, regardless of nanopore chemistry or sequencing depth (Tables S25 and S26). Plasmids were not detected in the extracted sequences from the assemblies containing *S*. Heidelberg, regardless of assembler, nanopore chemistry, or sequencing depth. In cases where the community contained *S*. Montevideo or Typhimurium, plasmid identification remained consistent across all assemblers. Notably, for the community with *S*. Heidelberg, none of the assemblers produced plasmid profiles that matched the reference genome, irrespective of nanopore chemistry or the number of reads. IncHI2 and IncHI2A were only identified in the extracted sequences from the Unicycler assembly using 1 million R10.4 reads. Interestingly, higher sequencing depths did not consistently lead to more accurate plasmid identification. For instance, in the community containing *S*. Typhimurium, the extracted sequences from the Flye assembly of 0.5 and 1 million R9.4.1 reads contained IncFIB(S) and IncFII(S), while the sequences from the Flye assembly of 2 million R9.4.1 reads failed to recover both plasmids. Additionally, although IncI1-I(Alpha) was not initially present in the Shasta assembly of 2 million R9.4.1 reads, it was subsequently detected in the extracted sequences from the assembly. Notably, the performance of R9.4.1 and R10.4 reads was comparable in terms of plasmid identification.

Regarding the community containing three serotypes, Flye outperformed other assemblers, with accurate results in all cases, followed by Raven and Shasta, regardless of nanopore chemistry or sequencing depth (Fig. 3; Tables S27 to S29). None of the Raven assemblies of R9.4.1 reads displayed plasmid profiles that were consistent with the reference genome, while higher sequencing depths (1 or 2 million reads) led to accurate results for the Raven assemblies of R10.4 reads. Interestingly, the Canu and Unicycler assemblies only produced accurate plasmid patterns when 0.5 million R9.4.1 reads and 1 million R10.4 reads were assembled, respectively. The Shasta assemblies of R9.4.1 reads generated precise plasmid identification at lower sequencing depths (0.5 and 1 million reads). In contrast, only the highest sequencing depth (2 million reads) resulted in accurate plasmid profiles for the Shasta assemblies of R10.4 reads. Similar to our observation of the community containing *S*. Heidelberg, the extracted sequences classified as *S*. Heidelberg in the community containing three serotypes did not carry any of the plasmids that were present in the reference genome. The extracted sequences classified as *S*. Montevideo from the Raven assemblies of 2 million R9.4.1 and R10.4 reads and the Shasta assembly of 0.5 million R9.4.1 reads carried IncI1-1(Alpha), which are consistent with the reference genome. In contrast, accurate plasmid identification was observed for the extracted sequences classified as *S*. Typhimurium from the Shata assemblies of R9.4.1 reads at all sequencing depths and the Unicycler assembly of 1 million R9.4.1 reads. Only the extracted sequences classified as *S*. Typhimurium from the Raven assembly of 2 million R10.4 reads showed plasmid patterns that were congruent with the reference genome.

#### Simulated surface water community and ZymoBIOMICS HMW DNA standard

Plasmids were absent in the reference genome of *P. aeruginosa* env207, as well as *S*. Chloleraesuis and *P. aeruginosa* in the ZymoBIOMICS HMW DNA standard. Therefore, they were not included in plasmid identification.

### MLST

#### Simulated fresh spinach community

**Fig 4:**
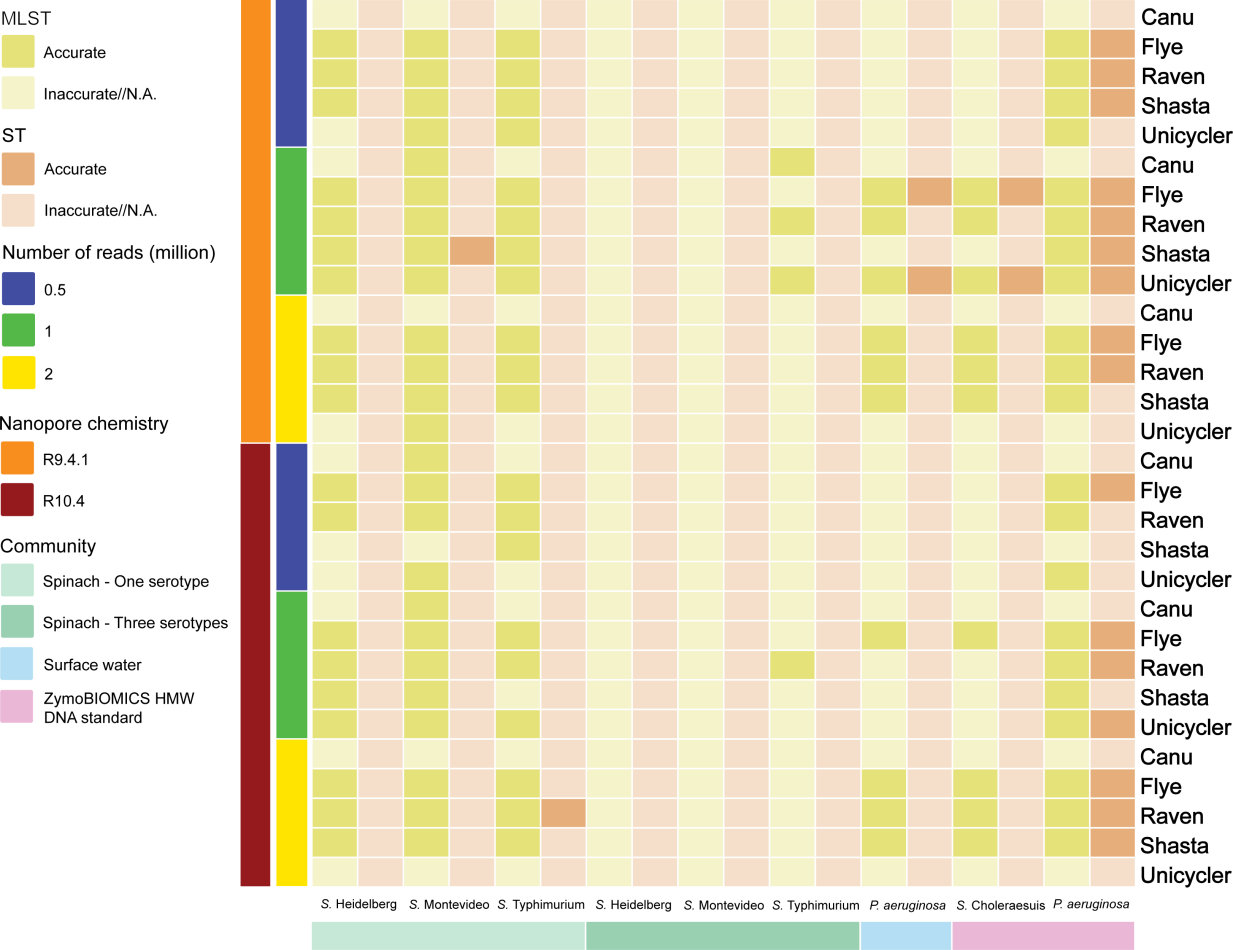
Multilocus sequence typing of the extracted sequences from metagenome assemblies.

When the community contained only one *S*. *enterica* serotype, precise MLST results were consistently obtained from the extracted sequences from all Flye and Raven assemblies, regardless of nanopore chemistry or sequencing depth, suggesting that Flye and Raven excelled among the assemblers (Fig. 4; Tables S30 to S32). They were closely followed by Shasta and Unicycler. Notably, Canu assemblies of the community containing *S*. Montevideo yielded accurate typing results only when 0.5 million R9.4.1 reads and 0.5 million or 1 million R10.4 reads were used for assembly. However, MLST results were not reported in any of the extracted sequences from the Canu assemblies of the community containing *S*. Heidelberg or Typhimurium. Accurate STs were exclusively provided for the extracted sequences from the Shasta assembly with 1 million R9.4.1 reads in the community containing *S*. Montevideo and the Raven assembly with 0.5 million R10.4 reads in the community containing *S*. Typhimurium. In contrast, when the community contained three serotypes, only the extracted sequences classified as *S*. Typhimurium from the Canu, Raven, and Shasta assemblies with 0.5 million R9.4.1 reads, along with the Raven assembly with 1 million R10.4 reads, provided accurate typing results (Tables S33 to S35). None of the extracted sequences yielded precise STs.

#### Simulated surface water community

Flye exhibited superior performance compared to other assemblers, closely followed by Raven and Shasta (Fig. 4; Table S36). Accurate MLST results were obtained from the extracted sequences from the Unicycler assemblies, but this was only achieved when 1 million R9.4.1 reads were used. On the other hand, none of the extracted sequences from the Canu assemblies were accurately typed. Specifically, precise STs were exclusively derived from the extracted sequences from the Flye and Unicycler assemblies when 1 million R9.4.1 reads were employed.

#### ZymoBIOMICS HMW DNA standard

The extracted sequences from the Flye assemblies consistently outperformed those generated by other assemblers (Fig. 4; Tables S37 and S38). Raven, Shasta, and Unicycler closely followed, regardless of the nanopore chemistry or sequencing depth used. Flye delivered accurate MLST results, with a single exception where the extracted sequences classified as *Salmonella* from the Flye assembly of 0.5 million R10.4 reads were inaccurately typed as *E. coli*. Conversely, none of the extracted sequences from the Canu assemblies were typed as *Salmonella* or *P. aeruginosa*. Among the assemblies that produced accurate MLST results for *P. aeruginosa*, only the extracted sequences from the Raven assembly of 0.5 million R10.4 reads, the Shasta assemblies of 2 million R9.4.1 reads and 1 million R10.4 reads, and the Unicycler assemblies of 0.5 million R9.4.1 and R10.4 reads did not yield an accurate ST.

### Serotyping

#### Simulated fresh spinach community

**Fig 5:**
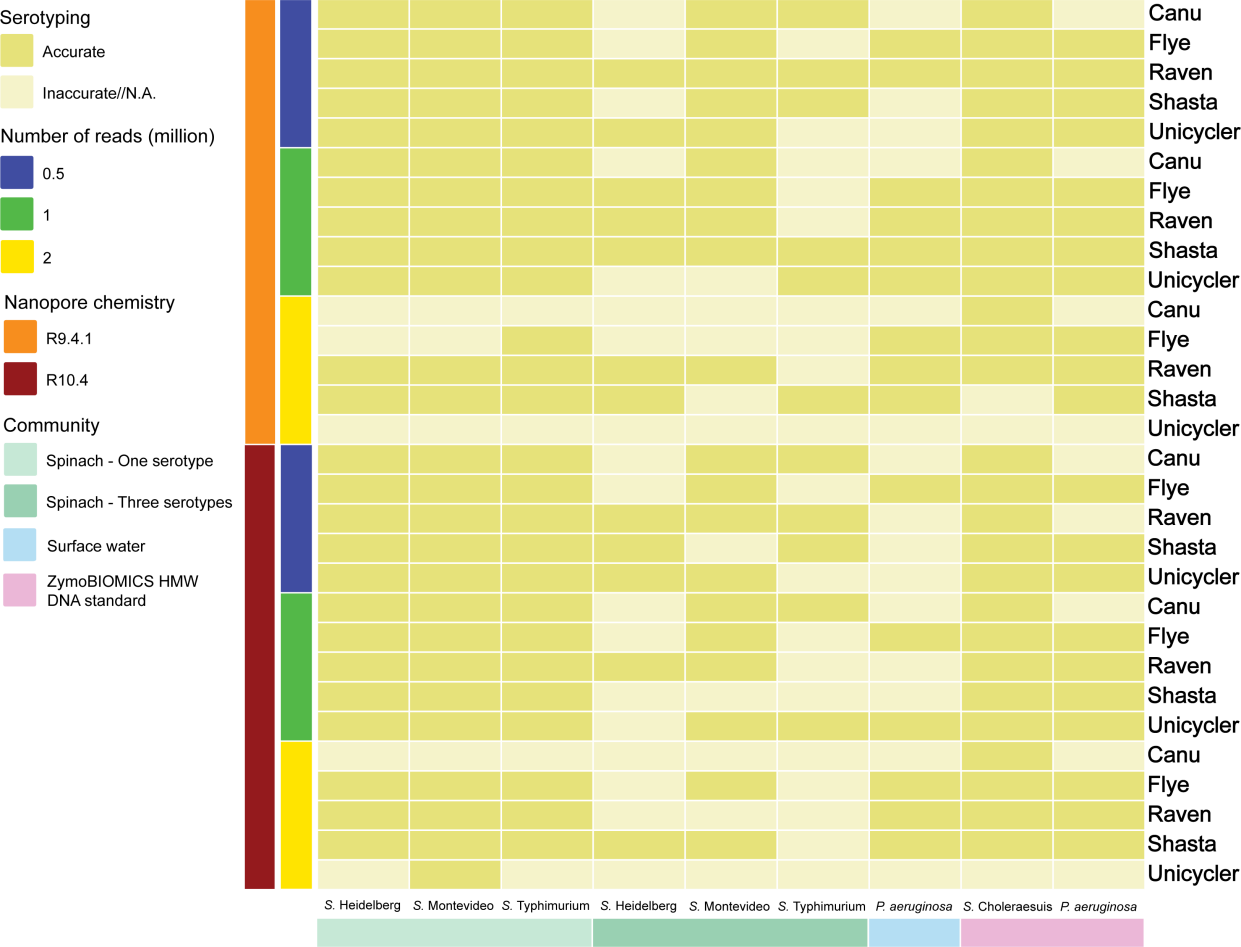
Serotyping of the extracted sequences from metagenome assemblies.

In scenarios where the community contained only one serotype, the extracted sequences were accurately assigned specific serotypes (Fig. 5; Tables S39 to S41). This remained consistent, unaffected by the choice of assembler, nanopore chemistry, or sequencing depth employed. There were only two noteworthy exceptions: the extracted sequences from the Flye assemblies with 2 million R9.4.1 reads in the community containing *S*. Heidelberg and Montevideo were inaccurately serotyped as *S*. Typhimurium and Heidelberg, respectively. This indicates that higher sequencing depth did not consistently improve *Salmonella* serotyping. Additionally, R10.4 appeared to be more effective in generating accurate results compared to R9.4.1.

When the community contained three serotypes, Raven emerged as the superior assembler, closely followed by Shasta (Fig. 5; Tables S42 to S44). Surprisingly, the performance of Flye was not as strong as when only one serotype was present in the community. Specifically, the extracted sequences classified as *S*. Typhimurium from the Flye assemblies failed to yield accurate results, irrespective of the nanopore chemistry or sequencing depth used. Unicycler and Canu demonstrated similar performance, although the extracted sequences classified as *S*. Heidelberg from the Canu assemblies were not accurately serotyped. No significant differences were observed between R9.4.1 and R10.4 sequencing chemistries nor among various sequencing depths.

#### Simulated surface water community

The extracted sequences from the Flye assemblies consistently received accurate results (Fig. 5; Table S45). The extracted sequences from the Raven and Shasta assemblies of R9.4.1 reads were more effective at producing precise results compared to R10.4 reads. In the case of the Unicycler assemblies, the extracted sequences were serotyped as O6 only when utilizing 1 million R9.4.1 and R10.4 reads. However, the Canu assemblies did not yield typable results, regardless of sequencing depth. Notably, higher sequencing depth led to more accurate serotyping.

#### ZymoBIOMICS HMW DNA standard

As for the extracted sequences classified as *Salmonella* and *P. aeruginosa*, they were consistently serotyped as Choleraesuis and O1, respectively, in all instances (Fig. 5; Tables S46 and S47). In contrast, the extracted sequences classified as *P. aeruginosa* from the Canu assemblies could not be serotyped.

### ARGs and point mutations

Notably, all contigs containing ARGs and point mutations in each assembly could be reliably identified when compared against the reference genomes using BLAST+. This enabled the determination of whether the ARGs and point mutations originated from the pathogens or other microorganisms, regardless of the specific community, assembler, nanopore chemistry, or sequencing depth employed.

#### Simulated fresh spinach community

**Fig 6:**
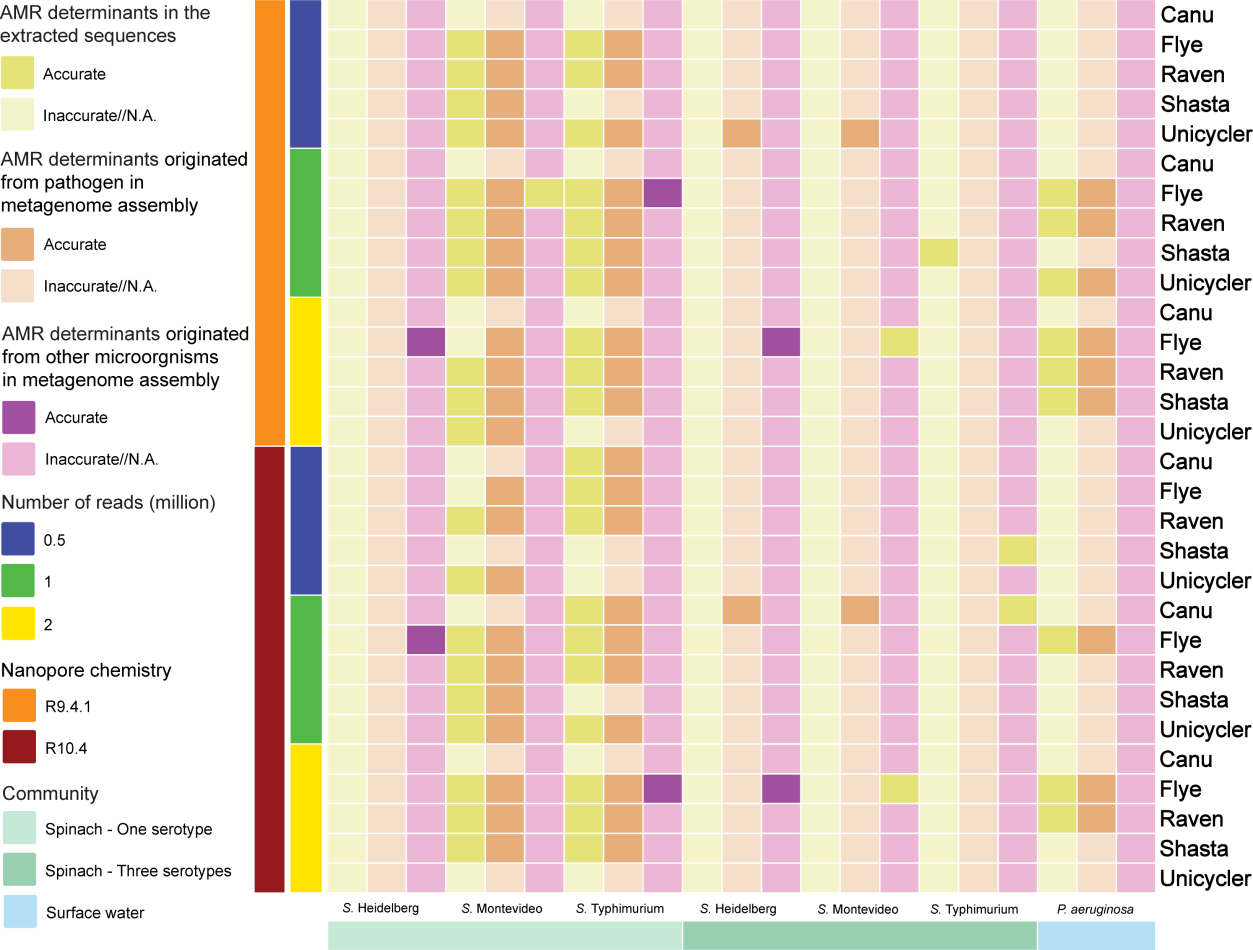
Antimicrobial resistance determinants (AMR genes and point mutations) in the extracted sequences from metagenome assemblies.

For the community containing *S*. Heidelberg, none of the extracted sequences consistently provided accurate AMR genotypes (Fig. 6; Tables S48 to S50). This remained consistent irrespective of the chosen nanopore chemistry or sequencing depth. However, in the Flye assemblies of 2 million R9.4.1 reads and 1 million R10.4 reads, the ARGs from other microorganisms were accurately identified, even though none of the assemblies carried ARGs or point mutations consistent with the reference genome.

In cases where *S*. Montevideo or Typhimurium was individually included in the community, Raven outperformed other assemblers, closely followed by Flye (Fig. 6). The accurate identification of ARGs from other microorganisms was only achieved with the Flye assemblies when 1 million R9.4.1 reads from the community containing *S*. Montevideo or Typhimurium were used. Canu was found to be the least effective assembler when only one serotype was present. It yielded accurate AMR genotypes for *S*. Typhimurium only in the extracted sequences of the assemblies of 0.5 and 1 million R10.4 reads in the community containing *S*. Typhimurium.

The presence of three serotypes led to decreased accuracy in identifying ARGs and point mutations (Fig. 6; Tables S51 to S53). The extracted sequences failed to capture ARGs and point mutations consistent with the reference genomes. There was only one exception where the extracted sequences classified as *S*. Typhimurium from the Shasta assembly of 1 million R9.4.1 reads provided an accurate AMR genotype. Notably, the Unicycler assembly of 0.5 million R9.4.1 reads for the community containing *S*. Montevideo was the sole assembly containing accurately identified ARGs and point mutations. Among the assemblies, only the Flye assemblies carried ARGs from other microorganisms consistent with the reference genomes.

#### Simulated surface water community

Flye outperformed other assemblers in terms of ARG and point mutation identification, closely followed by Raven (Fig. 6; Table S54). Canu, on the other hand, proved to be the least effective assembler, consistently yielding inaccurate AMR genotypes.

In most cases, R9.4.1 exhibited greater effectiveness than R10.4 in terms of ARG and point mutation identification. There were no significant differences observed among sequencing depths.

### Virulence genes

#### Simulated fresh spinach community

**Fig 7:**
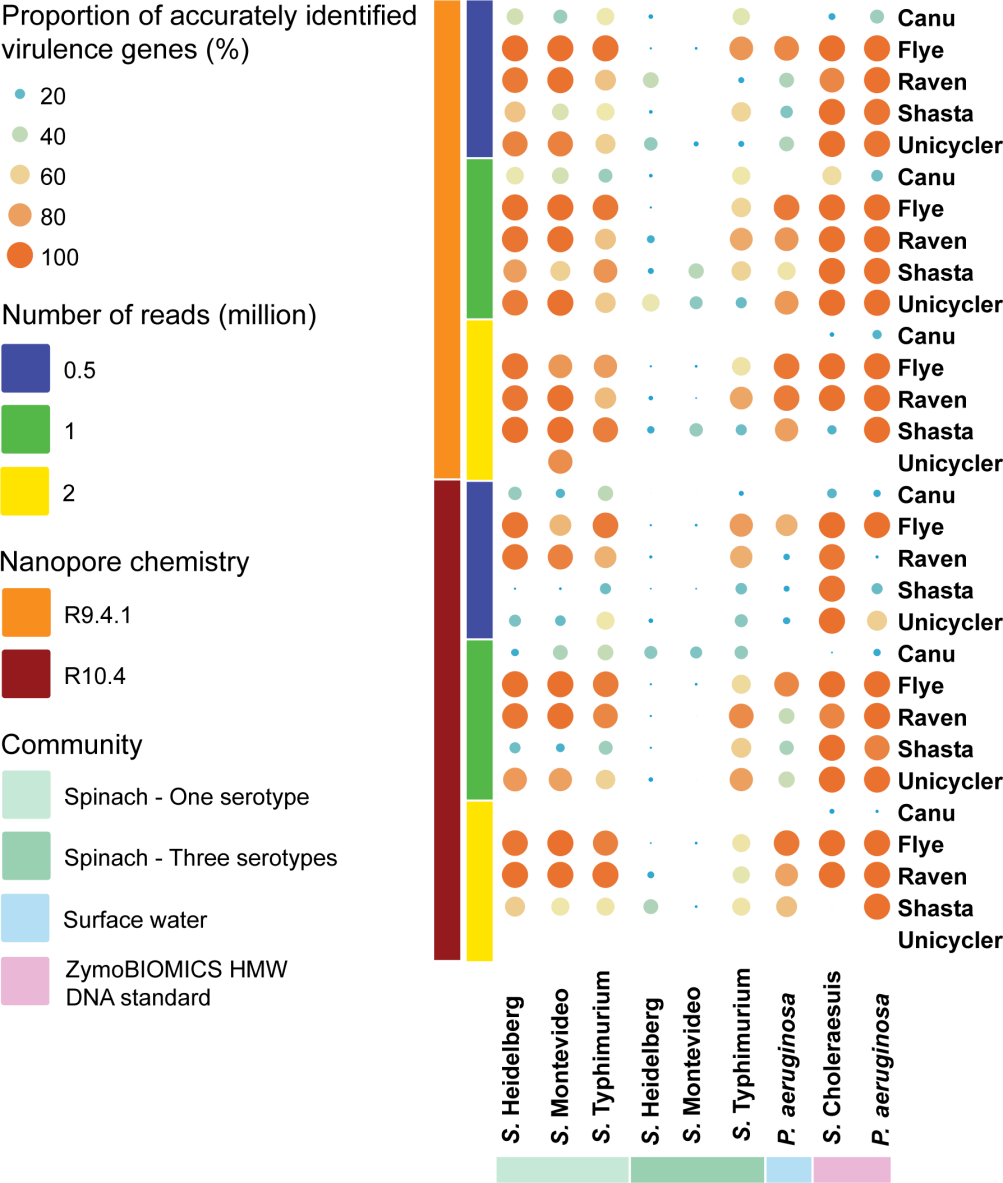
Proportions of accurately identified virulence genes in the extracted sequences from metagenome assemblies. The size of each circle is proportional to the proportions of accurately identified virulence genes in the extracted sequences from the metagenome assembly it represents.

In the case of the community containing a single *S. enterica* serotype, Flye and Raven performed similarly and demonstrated superior performance compared to other assemblers, followed by Shasta and Unicycler (Fig. 7; Tables S55 to S57). Canu consistently performed the worst among all assemblers. Increasing sequencing depths generally led to a more accurate identification of virulence genes in most instances. Notably, specific assemblies stood out, such as the extracted sequences from the Shasta assembly of 2 million R9.4.1 reads for the community containing *S*. Heidelberg, as well as the Flye assemblies of 0.5 and 1 million R9.4.1 reads, 1 and 2 million R10.4 reads, and the Shasta assembly of 2 million R9.4.1 reads for the community containing *S*. Montevideo, which harbored the same virulence genes as the reference genomes. Furthermore, R9.4.1 and R10.4 reads performed similarly in terms of virulence gene identification.

In the scenario where the community contained three serotypes, the extracted sequences classified as *S*. Heidelberg and Montevideo were consistently ineffective at identifying virulence genes, regardless of assembler, nanopore chemistry, or sequencing depth (Fig. 7; Tables S58 to S60). Conversely, for the extracted sequences classified as *S*. Typhimurium, Flye, Raven, and Shasta demonstrated similar performance. Notably, there were no significant differences observed among nanopore chemistries or sequencing depths.

#### Simulated surface water community

Flye exhibited a higher degree of effectiveness in identifying virulence genes compared to other assemblers, closely followed by Raven and Unicycler (Fig. 7; Table S61). Notably, the extracted sequences derived from the assemblies of R9.4.1 and R10.4 reads performed similarly. Moreover, higher sequencing depths led to improved performance by the assemblers. However, it is important to mention that none of the assemblers produced extracted sequences from the assemblies with virulence genes that were entirely consistent with the reference genome.

#### ZymoBIOMICS HMW DNA standard

When considering the extracted sequences classified as *Salmonella*, Flye, Raven, Shasta, and Unicycler demonstrated similar performance, while the extracted sequences from the Canu assemblies contained significantly fewer virulence genes (Fig. 7; Table S62). Notably, increasing sequencing depths did not result in more accurate identification. For the extracted sequences classified as *P. aeruginosa* from the assemblies of R9.4.1 reads, the performance of Flye, Raven, Shasta, and Unicycler showed no significant differences (Fig. 7; Table S63). However, when R10.4 reads were used for assembly, using 1 and 2 million reads led to more accurate identification compared to 0.5 million reads, particularly for Raven and Shasta. Remarkably, the extracted sequences from the Flye assemblies contained all virulence genes of *P. aeruginosa* for all sequencing depths. Additionally, the extracted sequences from the Flye, Raven, Shasta, and Unicycler assemblies provided virulence gene profiles consistent with the reference genome in some cases. In contrast, Canu was the least effective assembler in identifying virulence genes of *P. aeruginosa*, regardless of nanopore chemistry or sequencing depth. Furthermore, the extracted sequences classified as *Salmonella* and *P. aeruginosa* from the assemblies of R9.4.1 and R10.4 reads provided similarly accurate virulence gene profiles.

### SPIs

#### Simulated fresh spinach community

**Fig 8:**
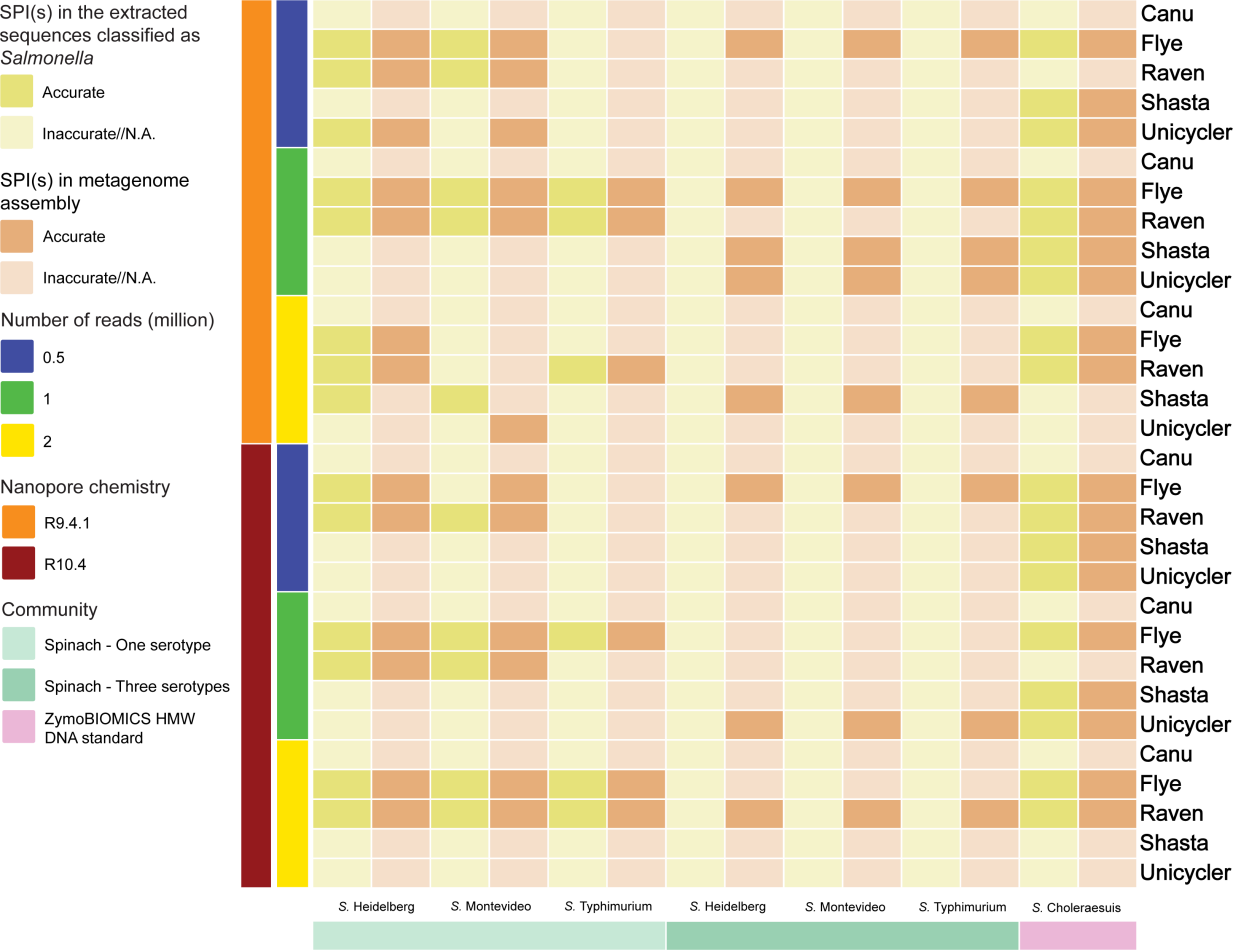
*Salmonella* pathogenicity islands in the extracted sequences classified as *Salmonella* from metagenome assemblies.

In the context of the community containing a single *S. enterica* serotype, Flye and Raven exhibited superior performance compared to other assemblers (Fig. 8; Tables S64 to S66). Conversely, the Canu and Shasta assemblies consistently failed to accurately identify SPIs in all instances. Interestingly, the Unicycler assemblies successfully captured all SPIs, particularly when assembling 0.5 million R9.4.1 reads for the community containing *S*. Heidelberg, and 0.5, 1, and 2 million R9.4.1 reads for the community containing *S*. Montevideo.

For the extracted sequences from the assemblies of the community containing a single serotype, Flye and Raven outperformed other assemblers when it came to SPI identification (Fig. 8; Tables S64 to S66). Meanwhile, Shasta and Unicycler demonstrated similar performance, showcasing accurate SPI profiles within the extracted sequences classified as *S*. Heidelberg and Montevideo. Specifically, the Shasta assemblies of 2 million R9.4.1 reads and the Unicycler assembly of 0.5 million R9.4.1 reads provided precise SPI profiles for these *Salmonella* serotypes. Conversely, none of the extracted sequences from the Canu assemblies exhibited SPI profiles consistent with the reference genomes.

For the community containing three serotypes, Canu consistently fell short in matching SPI profiles with the reference genomes, regardless of the nanopore chemistry or sequencing depth employed (Fig. 8; Tables S67 to S69). Flye emerged as the top performer, closely trailed by Shasta, Raven, and Unicycler. Accurate SPI identification was achieved in the Flye assemblies that utilized 0.5 and 1 million R9.4.1 reads, as well as 0.5 million R10.4 reads. On the other hand, Shasta and Raven provided accurate SPI profiles only with the use of 1 and 2 million R9.4.1 reads and 2 million R10.4 reads, respectively. However, none of the extracted sequences from these assemblies yielded precise SPI identification.

#### ZymoBIOMICS HMW DNA standard

The Flye and Unicycler assemblies successfully captured all SPIs present in the reference genome (Fig. 8; Table S70). In contrast, the Canu assemblies failed to show accurate SPI patterns. Raven and Shasta generated assemblies with accurate SPI profiles, utilizing 1 and 2 million reads and 0.5 and 1 million reads, respectively, regardless of the nanopore chemistry applied. The extracted sequences aligned consistently with the results obtained from these assemblies.

### Whole-genome phylogeny

**Fig 9:**
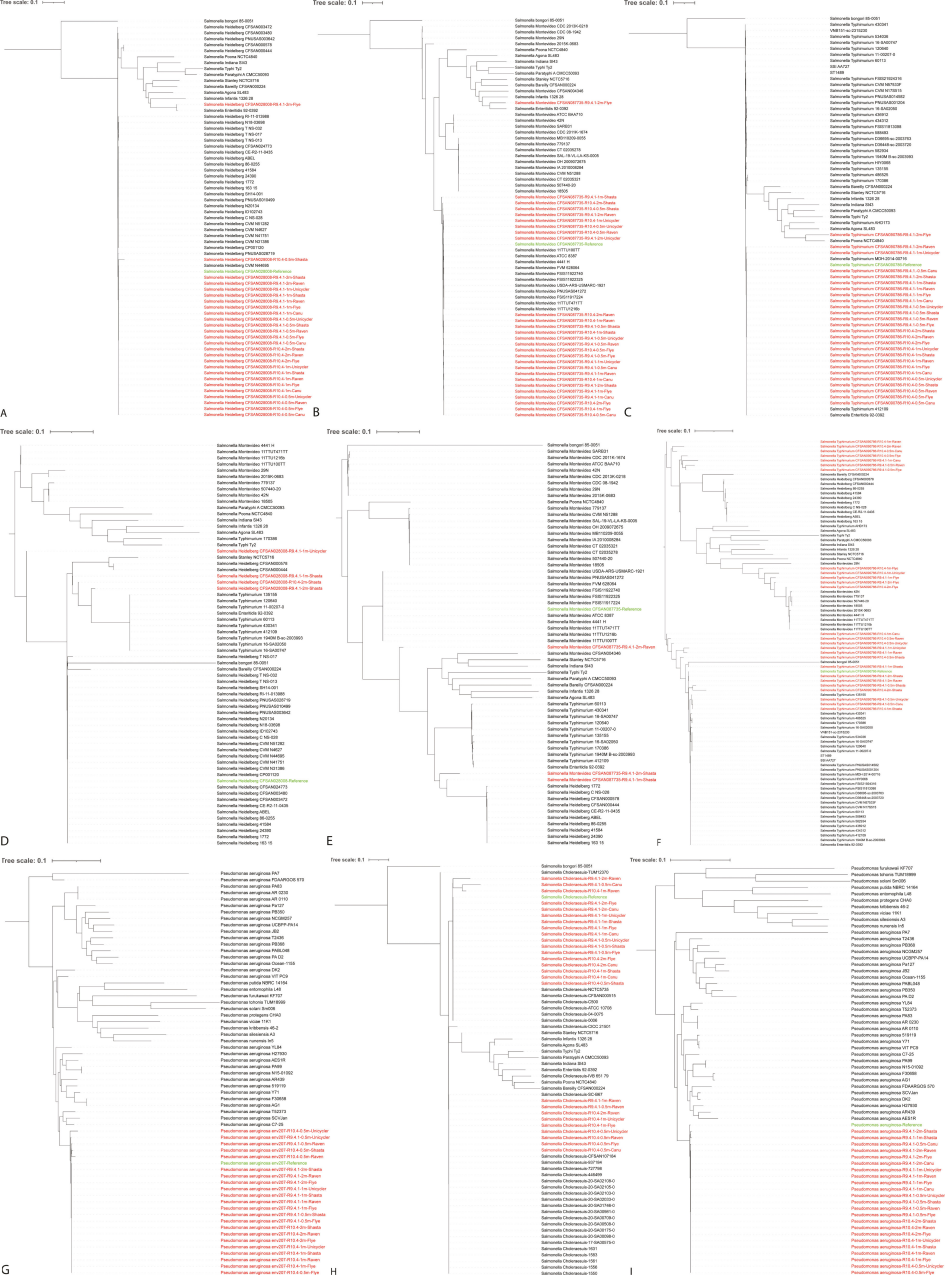
Whole-genome maximum-likelihood phylogenetic trees of the extracted sequences classified as *Salmonella* from the metagenome assemblies of the fresh spinach community containing *S*. Heidelberg (**A**), Montevideo (**B**), Typhimurium (**C**), those classified as *S*. Heidelberg (**D**), Montevideo (**E**), or Typhimurium (**F**) of the metagenome assemblies of the fresh spinach community containing a mixture of *S*. Heidelberg, Montevideo, and Typhimurium, those classified as *Pseudomonas aeruginosa* of the metagenome assemblies of the surface water community containing *P. aeruginosa* (**G**), and those classified as *Salmonella* (**H**) or *Pseudomonas aeruginosa* (**I**) of the metagenome assemblies of the ZymoBIOMICS HMW DNA standard compared to the reference genome and 30 *Salmonella* genomes of the same serotype and 10 *Salmonella* genomes of different serotypes or 30 *P*. *aeruginosa* genomes and 10 *Pseudomonas* genomes of different serotypes. For the fresh spinach community containing 3 serotypes, the extracted sequences for each serotype were also compared with 10 *Salmonella* genomes of two other serotypes. The scale bar stands for genetic distance. The extracted sequences are shown in red, while the reference genome is shown in green.+

Through our whole-genome phylogenetic analysis, which involved both the reference genomes and the extracted sequences, in most cases, the extracted sequences produced a phylogenetic tree topology that closely resembled that of the reference genomes. However, some extracted sequences were omitted from the analysis due to errors generated by CSI Phylogeny when dealing with sequences with inaccurate genome size information. As depicted in Fig. 9, the extracted sequences consistently formed a single monophyletic clade that was in alignment with the clade occupied by the reference genomes. This consistency held regardless of the nanopore chemistry or depth of sequencing applied, with a few exceptions. Specifically, in the case of the Flye assemblies of 2 million R9.4.1 reads for the fresh spinach community containing a single *S. enterica* serotype, the extracted sequences formed a dispersed paraphyletic clade relative to the major clade occupied by the reference genome and the extracted sequences from other assemblies (Fig. 9A through C). The performance of the extracted sequences from assemblies of the fresh spinach community containing three serotypes did not match the precision observed in the community containing a single serotype. Despite the challenges posed by the increased complexity of multiple serotypes (Fig. 9D through F), the extracted sequences from the assemblies for the fresh spinach community containing *S*. Typhimurium remained in close proximity to the reference genome. Overall, the extracted sequences from the Shasta assemblies exhibited the smallest distance from the reference genomes. Additionally, R9.4.1 reads generally outperformed R10.4 reads in most cases. Notably, there were no significant differences observed among sequencing depths. This underscores the potential utility of long-read metagenome assembly for accurate phylogenetic inference, as evidenced by the congruence between the phylogenetic topology of the reference genomes and the extracted sequences.

## DISCUSSIONS

While assessing a metagenome assembly algorithm, it is important to recognize that assembly quality statistics should not be the sole determining factor. In our current study, we have placed significant emphasis on the depth of metagenomic and genomic information extracted from the metagenome assembly for subsequent analyses. Many of the long-read assembly algorithms currently available were originally designed for single-genome assembly, focusing on selecting high-frequency *k*-mers. However, in a metagenomic context, this strategy tends to favor high-abundance species, potentially leaving low-abundance species with fewer or no *k*-mers, thus resulting in failed assembly of their reads. Therefore, the ideal assembly algorithm should combine global *k*-mer counting with an analysis of local *k*-mer distributions. In our study, we noted that Flye, which incorporates a scalable *k*-mer selection algorithm called metaFlye, proved to be the most effective assembler for enhancing the metagenomic identification of bacterial pathogens. This assembler excels in addressing the challenges presented by uneven bacterial composition and intra-species heterogeneity in metagenome assembly. Our findings are in-line with previous research conducted by Kolmogorov et al. (29), the developers of metaFlye. They demonstrated significant improvements of metaFlye over Canu and miniasm, particularly highlighting that metaFlye generated more contiguous contigs and successfully captured a greater number of plasmids.

While we acknowledge the inherent limitations of simulated reads, it is crucial to highlight that our simulated data sets were generated based on R9.4 and R10.4 error models derived from real data sets published by Sereika et al. (17). This approach aimed to bridge the gap between simulated and real-world scenarios, enhancing the relevance of our simulated data. In our study, we incorporated both simulated and real data sets to offer a comprehensive evaluation. Mock communities such as the ZymoBIOMICS Microbial Community, which contain known reference genomes, serve as valuable benchmarks, offering realistic features relevant to Oxford Nanopore long reads. However, it is crucial to recognize that these communities may not fully mimic the complexity of actual food or environmental microbiota. Despite the relatively simplified nature of the ZymoBIOMICS Microbial Community Standard compared to simulated communities, our research underscores the consistency observed in assembly results. This consistency reinforces the robustness of our findings and supports the inclusion of simulated data sets to provide a comprehensive evaluation of assembly algorithms.

In our prior investigation, when we evaluated short-read assembly algorithms using identical fresh spinach and surface water communities (53), no draft MAGs were obtained. Short-read assemblies yielded contigs with considerably less contiguity and a higher level of fragmentation compared to long-read assemblies. The lower contiguity observed in short-read assemblies also resulted in highly fragmented extracted sequences classified as *Salmonella* and *P. aeruginosa*. This, in turn, hindered downstream genomic analyses, particularly those reliant on high-contiguity genomes for identifying the locations and structures of genes and mobile genetic elements. Although short-read assembly algorithms have made progress in enhancing the resolution of whole-genome phylogenetic analysis, the extraction of complete genomic data from the assembly remains a difficult task, as revealed by our previous study (53). In contrast, our current study revealed that in certain instances, assembling Oxford Nanopore long reads led to exceptionally high-quality assemblies and yielded contiguous extracted sequences classified as *Salmonella* and *P. aeruginosa*, particularly when employing the Flye assembler. This heightened level of contiguity significantly enhanced the precision of subsequent genomic analyses. For instance, in the present study, when examining the contigs containing ARGs within each assembly, we found that all of them could be accurately matched to the reference genomes. This allowed us to discern whether the ARGs originated from the pathogens or other microorganisms in the communities. MAGs provide a granular view, enhancing our ability to pinpoint specific pathogenic strains within complex microbial populations. The use of MAGs to identify and characterize bacterial strains at the genomic level is also crucial for understanding variations in antimicrobial resistance, virulence, and other key traits. MAGs empower researchers to investigate strain-level dynamics, offering insights into the genetic makeup of pathogens present in food and environmental samples.

The effectiveness of Kraken 2, with its classification algorithm, reference taxonomies, and standardized database, in classifying and extracting metagenomic reads in our study has raised some concerns. This is particularly notable when dealing with sequence extraction from assemblies of bacterial communities, especially when multiple *S. enterica* serotypes belonging to the same O group were involved. For instance, in cases where three serotypes were present within the fresh spinach community, we observed variations in the accuracy of serotyping among the extracted sequences. Interestingly, the extracted sequences classified as *S*. Montevideo were more reliably identified than those classified as *S*. Heidelberg and Typhimurium. One plausible explanation for this divergence is that Heidelberg and Typhimurium, both belonging to group O:4, share closer genetic markers. Kraken 2 operates by matching short DNA sequences (*k*-mers) from the query sequences to a pre-built reference database. In cases where different serotypes are highly similar, the shared *k*-mers may not provide enough discriminatory information for accurate identification. This can result in ambiguities and errors in classification. Additionally, we noted that even when only one serotype was present, the extracted sequences classified as *S*. Typhimurium exhibited higher quality compared to those classified as *S*. Heidelberg and Montevideo. The accuracy of classification relies heavily on the availability of reference sequences in the database. This discrepancy could be attributed to the larger number of available *S*. Typhimurium genomes (over 30,000) on NCBI in contrast to the fewer genomes available for *S*. Heidelberg and Montevideo (less than 5,000). The accuracy of the extracted sequences primarily hinges on the pre-built *k*-mer database of Kraken 2 and the taxa that can be rapidly matched to *k*-mers detected in each assembly. A more extensive reference database for a particular serotype can enhance the ability of Kraken 2 to accurately identify and classify sequences associated with that serotype. As the whole-genome databases on NCBI continue to expand, the reference sequences available for aligning sequences and mapping *k*-mers will naturally evolve to accommodate these changes.

We sought to explore how R9.4.1 reads differ from R10.4 reads generated by the newly developed nanopore chemistry in terms of the use of metagenome assembly to improve the metagenomic identification of bacterial pathogens. Not surprisingly, we did observe some noteworthy nanopore chemistry-specific variations in our data. However, we noticed that despite the higher quality of R10.4 reads, the assemblies of R9.4.1 reads appeared to have an advantage over the assemblies of R10.4 reads in most cases regarding the metagenomic identification of bacterial pathogens. Conflicting results, however, were obtained in the research by Sereika et al. (17), who observed an improvement in the accuracy of the Flye assembly when transitioning from R9.4.1 to R10.4. Nevertheless, in their study, there has been a lack of comprehensive exploration of alternative long-read assembly methods for metagenome assembly or the extracted sequences from the assemblies for subsequent genomic analyses. It should be noted that in our study, R9.4.1 reads exhibited longer lengths despite lower quality compared to R10.4 reads (Table S3; Fig. S1), which could influence assembly outcomes. Longer reads are generally advantageous for resolving complex genomic regions and aiding in the assembly of repetitive elements (54). However, the trade-off between read length and quality is a complex interplay, and the downstream effects on assembly outcomes also vary. Longer reads may improve the contiguity of contigs, whereas lower-quality reads could introduce errors. Conversely, shorter reads with higher quality may produce more accurate contigs but could pose challenges in resolving repetitive regions. Further studies with a more focused investigation into the nuanced effects of read characteristics on assembly outcomes can contribute to a deeper understanding of these dynamics. Moreover, newer sequencing technologies often require adjustments in assembly algorithms to fully utilize their potential. One plausible explanation of our observations could be that if the algorithms used for metagenome assembly were primarily developed or optimized for the R9.4.1 chemistry, it might not be fully adapted to handle R10.4 data optimally. Different nanopore chemistries may have distinct error profiles. While newer chemistries aim to reduce errors, they might introduce new types of errors or variations that assembly algorithms are not yet equipped to handle. Therefore, older chemistries might have had their errors characterized and addressed more effectively. It is worth noting that the field of nanopore chemistry is highly dynamic, with continuous improvements and updates. As a result, the relative performance of different nanopore chemistries may change over time as assembly algorithms are updated and refined to harness the strengths of each new technology.

Meanwhile, we also acknowledge the potential influence of library preparation methods on the assembly quality, a critical aspect often intertwined with sequencing outcomes. Specifically, the choice of library preparation kits, such as SQK-LSK109 for the R9.4.1 flow cell and SQK-LSK112 for the R10.4 flow cell, can impact several facets of the metagenomic sequencing process. Our study sought to faithfully replicate the experimental conditions outlined in Sereika et al. (17) who utilized R9.4.1+SQK-LSK109 and R10.4 + SQK-LSK112. While our study focuses on evaluating the performance of long-read assembly algorithms, it is imperative to recognize the interconnected role of library preparation methods in shaping subsequent sequencing landscape. Future investigations should systematically explore the impact of various library preparation kits on assembly quality, providing a more comprehensive understanding of the factors influencing downstream analyses. Moreover, we recognize the importance of basecalling models in Oxford Nanopore sequencing. In subsequent studies, we plan to systematically explore the impact of basecalling models on assembly accuracy to provide a more thorough understanding of the interplay between basecalling choices and metagenome assembly outcomes.

Currently, there is no universally accepted standard for determining the necessary sequencing depth to ensure accurate metagenomic identification. Additionally, there is a lack of comprehensive knowledge regarding the specific sequencing depth requirements for different nanopore chemistries and bacterial pathogens. When working with complex metagenomic samples, obtaining an adequate sequencing depth often generates a substantial volume of data. As anticipated, increasing sequencing depths generally leads to more precise metagenomic identification. Nevertheless, it is essential to note that there were situations in which an excessively large number of reads introduced additional errors. For instance, in the fresh spinach community with *S*. Typhimurium, sequences extracted from the Flye assembly of 0.5 million and 1 million R9.4.1 reads included IncFIB(S) and IncFII(S) plasmids, whereas the sequences from the Flye assembly of 2 million R9.4.1 reads did not successfully capture both plasmids. Increasing sequencing depths comes with its own set of challenges. It elevates the risk of introducing sequencing errors, particularly if these errors are systematic and inherent to the nanopore chemistry used. Such errors can proliferate throughout the entire assembly and analysis process, potentially giving rise to spurious variations and leading to incorrect identifications. Moreover, with the augmentation of sequencing depth, the likelihood of encountering chimeric reads also rises, where sequences from different organisms are merged (55). Chimeric reads can result in misassemblies and misidentifications, complicating the accurate assignment of sequences to their correct source organisms. In complex metagenomic samples teeming with diverse organisms, the abundance of data generated at high sequencing depths can make it more challenging to distinguish between closely related species. This can lead to ambiguous or erroneous identifications. Furthermore, metagenomic samples often exhibit a non-uniform distribution of organisms, with some species being more prevalent than others (56). When sequencing depth is increased, there is a risk of overrepresenting the more abundant species, making it harder to detect rarer yet crucial organisms. To mitigate these issues and harness the advantages of increasing sequencing depth, researchers should be diligent in their choice of assembly algorithms. Striking the right balance between sequencing depth, sample complexity, and research objectives is vital for achieving precise metagenomic identification while minimizing the introduction of unwarranted errors. Although our current study employed a random sub-sampling strategy for different sequencing depths, we acknowledge the importance of considering the sequencing time in future studies and its potential influence on read characteristics due to the biological characteristics of sequencing pores. This approach will provide valuable insights into the dynamics of sequencing time and its effects on read properties, contributing to a thorough understanding of the interplay between chronological order and read accuracy.

Our study demonstrates the potential of using the extracted sequences from metagenome assemblies for accurate phylogenetic inference, as evidenced by the consistent phylogenetic topology observed between the reference genome and the extracted sequences. However, it is important to exercise caution when interpreting these results, particularly when dealing with closely related strains. In routine clinical and surveillance contexts, we do not propose that phylogenetic analysis can currently rely solely on the extracted sequences. Instead, we believe that information from these assemblies can complement other currently used identification methods. It is expected that ongoing enhancements to long-read assembly algorithms will systematically improve assembly quality to the extent that accurate phylogenetic inferences can be achieved using the extracted sequences.

It is essential to acknowledge the potential impact of post-assembly polishing on improving assembly outcomes. In future studies, it would be prudent to explore the effects of polishing on assembly outcomes, especially given the reported improvements with R9.4.1 reads but not necessarily with R10.4 reads (57). As assembly algorithms and nanopore chemistries continue to advance, understanding the nuanced impact of polishing on assemblies becomes increasingly pertinent. Future work will delve into the optimization of polishing steps for specific nanopore chemistries, guiding researchers in tailoring their analysis for improved metagenomic pathogen identification. Meanwhile, while our current study provides valuable insights into the efficiency of assemblers and the benefits of Oxford Nanopore long reads for downstream analyses without any contamination, future investigations may explore avenues to reduce contamination in MAGs obtained from complicated microbiomes. Addressing contamination concerns could further enhance the reliability and accuracy of metagenomic and genomic analyses. Exploring advanced bioinformatic strategies or refining assembly methodologies may contribute to minimizing contamination in MAGs, thereby advancing the application of Oxford Nanopore long reads in metagenomic research. Future studies dedicated to optimizing MAG quality metrics will contribute to a more comprehensive understanding of the capabilities and limitations of long-read assembly algorithms.

The simulated data set used in our study leveraged *Salmonella* as a representative of the Enterobacteriaceae family. We acknowledge that such high abundances may introduce biases during MAG construction, potentially favoring the representation of highly abundant strains. Recognizing the importance of understanding and mitigating potential biases, our future research will delve into the impact of relative abundance on downstream pathogen identification. This consideration aligns with the evolving landscape of metagenomic research, emphasizing the need to scrutinize and refine methodologies to ensure robust and unbiased outcomes. Our evaluation of assemblers was conducted using default parameters and recommended settings, but potential enhancements in these parameters before implementation could improve microbiome reconstruction accuracy. It is vital to highlight that while our study offers valuable insights into assembler performance, we understand the time-sensitive nature of this research, and continuous reassessment is essential due to the rapid evolution of assembly algorithms and nanopore chemistries. Based on our findings, we urge researchers to carefully consider their research objectives and metagenomic sample characteristics when selecting suitable assemblers. We also recognize the importance of extending our research to include diverse and complex microbial communities such as those found in soil and gut. These communities present unique challenges and opportunities for metagenomic analyses, and their inclusion would enhance the robustness and generalizability of our findings. We, therefore, plan to extend our investigations to encompass a broader array of complex communities in future studies. By incorporating diverse microbial ecosystems, we aim to contribute to the improvement of MAGenie that can be standardized across various communities, ultimately broadening the applicability of our methodology. Additionally, we acknowledge the need for future evidence-based research comparing third-generation sequencing technologies like Oxford Nanopore and PacBio, as well as their combination with Illumina sequencing, for the metagenomic identification of bacterial pathogens. Such research will aid in determining the most appropriate assembly algorithms for these technologies. While MAGenie currently includes a series of interconnected steps with publicly available tools utilized sequentially, it will be integrated into a cohesive and user-friendly platform after benchmarking it extensively using a variety of communities, particularly incorporating established tools such as bettercallsal previously developed by the FDA (58) for *Salmonella* serotyping within a metagenomic context to identify taxonomy IDs prior to using MAGenie to extract draft MAGs. This integration will enhance the robustness of MAGenie, ensuring its suitability for future applications in food safety research and pathogen surveillance.

### Conclusions

Recent advancements in long-assembly algorithms have harnessed the power of Oxford Nanopore sequencing to reconstruct more contiguous draft MAGs from complex metagenomic samples compared to Illumina sequencing. Our study demonstrates more accurate metagenomic and genomic analyses achieved with the Flye assemblies, addressing the challenges posed by the high error rates associated with this technology. These findings underscore the suitability of Oxford Nanopore sequencing, coupled with long-read assembly algorithms for assembling complex bacterial communities, taxonomic classification, and extracting draft MAGs from metagenome assemblies. This work contributes to the future standardization of MAGenie for long-read sequencing data, particularly aiding researchers in identifying bacterial pathogens in a metagenomic context. As metagenome assembly algorithms continue to evolve, ongoing assessment of these tools is essential for improving metagenomic identification capabilities.

## Data Availability

Simulated data sets were deposited into the Sequence Read Archive (SRA) database on NCBI under the BioProject accession number PRJNA1090476.

## SUPPLEMENTAL MATERIAL

Fig. S1 spectrum.00117-24-s0001.docx Read length distributions of Oxford Nanopore.

Supplemental material spectrum.00117-24-s0002.xlsx Tables S1-S70.

ASM does not own the copyrights to Supplemental Material that may be linked to, or accessed through, an article. The authors have granted ASM a non-exclusive, world-wide license to publish the Supplemental Material files. Please contact the corresponding author directly for reuse.
