# Supplementary material for: Advancing metagenome-assembled genome-based pathogen identification: unraveling the power of long-read assembly algorithms in Oxford Nanopore sequencing
Source: Microbiol Spectr. 2024 Apr 30;12(6):e00117-24. doi: 10.1128/spectrum.00117-24 (PMC11237517; doi:10.1128/spectrum.00117-24)
Supplement: Fig. S1 — Read length distributions of Oxford Nanopore. [file spectrum.00117-24-s0001.docx]

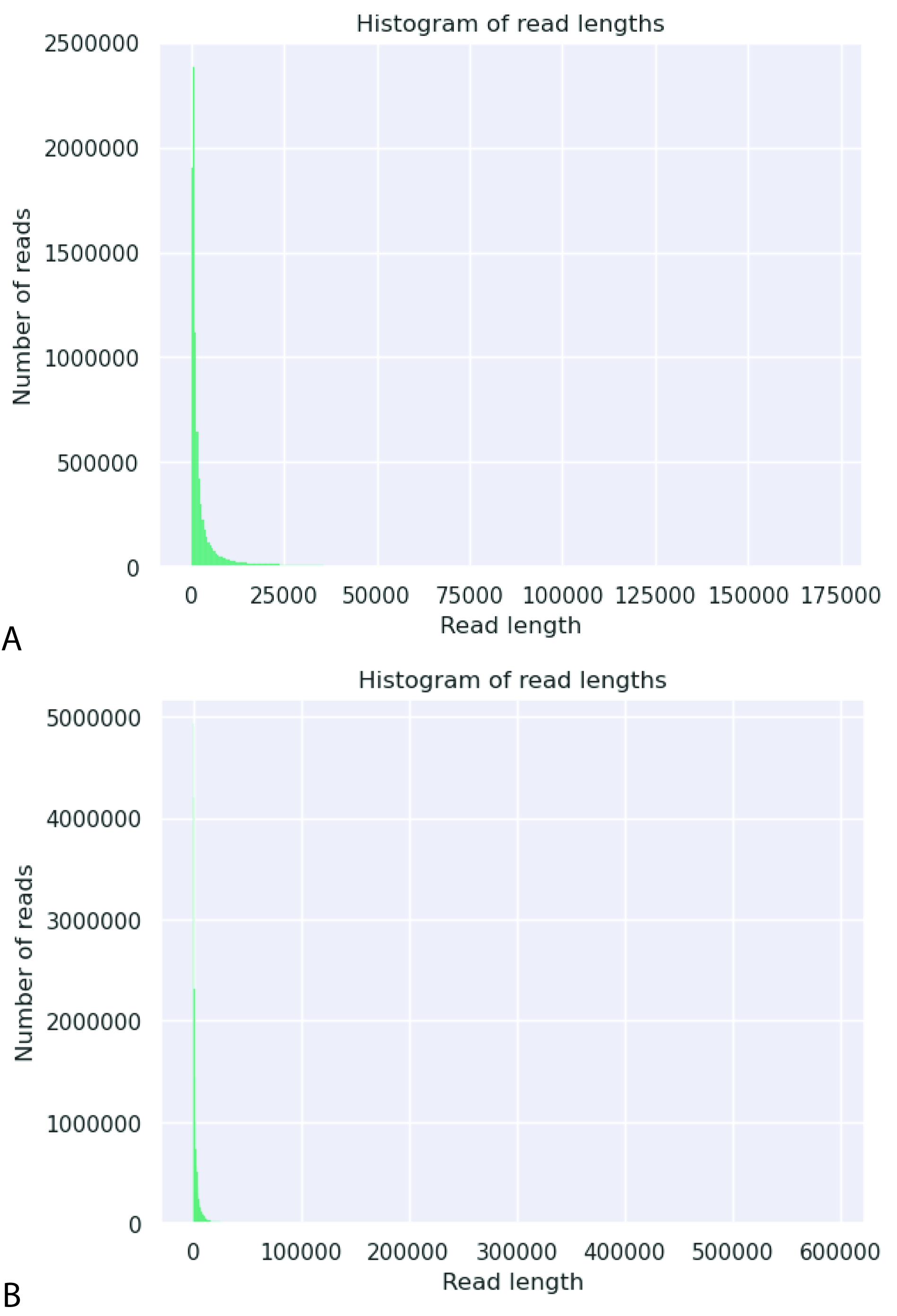


**Figure S1** Read length distributions of Oxford Nanopore long reads generated with the R9.4.1 (A) and R10.4 (B) flow cells.
